# Long Term Running Biphasically Improves Methylglyoxal-Related Metabolism, Redox Homeostasis and Neurotrophic Support within Adult Mouse Brain Cortex

**DOI:** 10.1371/journal.pone.0031401

**Published:** 2012-02-08

**Authors:** Stefano Falone, Antonella D'Alessandro, Alessandro Mirabilio, Giacomo Petruccelli, Marisa Cacchio, Carmine Di Ilio, Silvia Di Loreto, Fernanda Amicarelli

**Affiliations:** 1 Department of Basic and Applied Biology, University of L'Aquila, L'Aquila, Italy; 2 Department of Biomedical Sciences, University “G. d'Annunzio”, Chieti Scalo, Italy; 3 Department of Basic and Applied Medical Sciences, University “G. d'Annunzio”, Chieti Scalo, Italy; 4 Institute of Translational Pharmacology, National Research Council, L'Aquila, Italy; Universidad Europea de Madrid, Spain

## Abstract

Oxidative stress and neurotrophic support decline seem to be crucially involved in brain aging. Emerging evidences indicate the pro-oxidant methylglyoxal (MG) as a key player in the age-related dicarbonyl stress and molecular damage within the central nervous system. Although exercise promotes the overproduction of reactive oxygen species, habitual exercise may retard cellular aging and reduce the age-dependent cognitive decline through hormetic adaptations, yet molecular mechanisms underlying beneficial effects of exercise are still largely unclear. In particular, whereas adaptive responses induced by exercise initiated in youth have been broadly investigated, the effects of chronic and moderate exercise begun in adult age on biochemical hallmarks of very early senescence in mammal brains have not been extensively studied. This research investigated whether a long-term, forced and moderate running initiated in adult age may affect the interplay between the redox-related profile and the oxidative-/MG-dependent molecular damage patterns in CD1 female mice cortices; as well, we investigated possible exercise-induced effects on the activity of the brain derived neurotrophic factor (BDNF)-dependent pathway. Our findings suggested that after a transient imbalance in almost all parameters investigated, the lately-initiated exercise regimen strongly reduced molecular damage profiles in brains of adult mice, by enhancing activities of the main ROS- and MG-targeting scavenging systems, as well as by preserving the BDNF-dependent signaling through the transition from adult to middle age.

## Introduction

Aging is associated with the accumulation of reactive oxygen species (ROS)-damaged biomolecules and this phenomenon may be due to either an increased production of ROS or to a decreased efficiency of antioxidant defenses; this condition may facilitate the onset of oxidative stress, thus promoting structural and functional alterations [1]–[3]. The central nervous system (CNS) is highly susceptible to the oxidative stress and to senescence, mainly due to the high oxidative metabolism and to the poor self-repairing capacities of post-mitotic neuronal populations; in addition, high levels of redox-active metal ions, relatively low antioxidant defenses, and the presence of membranes highly prone to peroxidative attack also account for the high vulnerability of the brain towards oxidative damage [4], [5]. Coherently, many studies have observed in aged brains increased levels of oxidatively-modified lipids and proteins, and these molecular alterations are thought to play major roles in the pathogenesis of several age-related neurodegenerations [5].

Brain senescence and many age-related diseases are also commonly associated with increased levels of glycative-modified macromolecules. Methylglyoxal (MG), a pro-apoptotic and pro-oxidant dicarbonyl compound, is one of the main responsible for glycating reactions towards proteins and lipids within aged tissues, where MG reacts predominantly with arginine residues to form, among other derivatives, hydroimidazolones and arg-pyrimidine [6]–[8]. As MG is endogenously formed mainly as a byproduct of carbohydrate and lipid metabolism, organisms have developed the glyoxalase system, an ubiquitous detoxification pathway protecting cells from MG-related cytotoxicity [9]. Many researchers have reported age-dependent increases in the levels of proteins structurally and functionally modified by MG, and this is essentially due to either a reduced efficiency of the glyoxalase system or to an increase of its glycolytic precursors [10]. MG impairs cell redox homeostasis by inhibiting glyoxalases and by depleting the intracellular pool of reduced glutathione (GSH), which is an essential co-factor for glyoxalase 1 and for crucial antioxidant enzymes, such as the glutathione peroxidase (GPX) [11], [12]. As discussed by Kuhla and colleagues [13], MG cytotoxicity may play a very important role in brain aging. Our group has demonstrated that MG is strongly toxic to nervous cells, in which it triggers both a pro-oxidant shift in the cellular milieu and pro-inflammatory responses [14]. In addition, our group has shown that MG alters the expression levels of both brain derived neurotrophic factor (BDNF) and its high affinity tyrosinekinase B receptor (TrkB), thus eventually affecting important neuronal functions [15].

Although physical exercise promotes ROS overproduction [16], a sedentary lifestyle is becoming increasingly known as an accelerating factor towards unhealthy aging [17]. This apparent paradox could be explained by taking into account that the habitual exposure to exercise bouts triggers hormetic responses by inducing many biochemical and molecular adaptations, such as the enhancement of crucial neurotrophic support and antioxidant defenses, thus improving the resistance towards oxidative stress [18]–[20]. Several authors have also reported that a regular and moderate physical exercise reduces the age-dependent cognitive decline and preserves abilities in learning and memory tasks [21]–[23]. These findings encourage a growing number of sedentary individuals of adult and mature age to initiate physical exercise through which weight status, cardiovascular function, metabolic and brain health could be significantly controlled or preserved.

However, whereas hormetic responses induced by exercise initiated in youth have been broadly investigated, the effects of chronic and moderate exercise begun in adult and mature age on biochemical hallmarks of very early senescence in mammal brains have not been extensively studied, and only sporadic literature is available [21], [24]. Moreover, despite the large research interest in this field, no scientific evidence has been provided so far about the role of physical exercise in regulating the accumulation of brain MG-related molecular damage. Hence, physiological changes and responses induced by exercise within CNS might be explored by conceiving an interplay among MG-related metabolism and damage, antioxidative pathways and neurotrophic support. Indeed, the BDNF-dependent signaling pathways, such as the phosphorilated cAMP response element binding (p-CREB) transcriptional activator and the phosphorilated TrkB have been suggested to be involved in positive adaptations triggered by exercise within brain [19], [25]–[27].

On this basis, we performed a time-course analysis aimed at assessing whether a program of moderate and regular exercise could affect the interplay between redox-related profiles and oxidative-/MG-dependent molecular damage patterns in brain cortices of mice undergoing the transition from adult to middle age. Several age-dependent cognitive declines have been reported during this biological time period [28], which is roughly similar to the transition through the mature age in humans [29], therefore this age range may be adequate to investigate exercise-dependent central effects. In addition, we investigated the possible interactions of the BDNF-activated pathway with the above mentioned processes. Our experimental design involved non spontaneous physical activity since some preeminent authors have argued that forced exercise protocols are more similar to human exercise practise and less likely biased depending on genetic differences between good and poor runners [30], [31].

## Results

### Effects of age and exercise on ponderal state and food intake

No significant main effect of age was detected on body weight or food consumption (not shown). Treadmill running induced no significant alterations in ponderal state or appetence behaviour (not shown).

### Effects of age and exercise on oxidative damage and antioxidant/redox status

**Figure 1:**
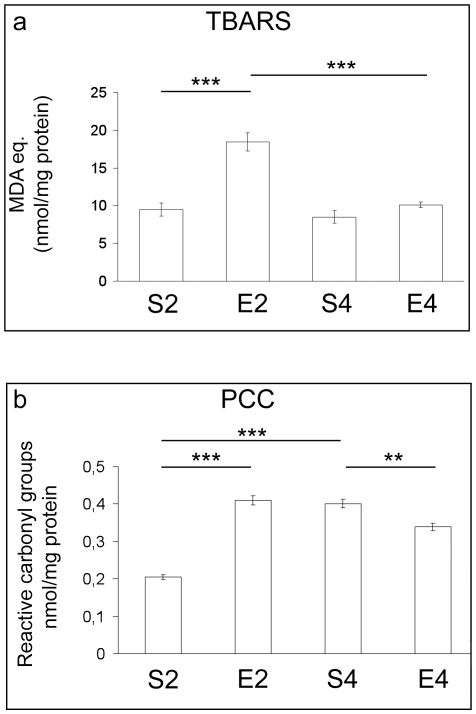
Brain molecular damage of mice undergoing a long-term moderate treadmill running. Thiobarbituric acid-reactive substances (TBARS, panel a) levels and protein carbonyl content (PCC, panel b) in cortices of mice undergoing a two- or four-month moderate and regular treadmill-based exercise program (E2 or E4, respectively); age-matched sedentary animals (S2, S4) were used as controls (n = 6 per group). No significant age-dependent change was observed in cortical TBARS concentrations (S4 vs S2), while two months of exercise elevated significantly TBARS amounts (E2 vs S2). Four-month exercised and unexercised mice showed similar TBARS levels (E4 vs S4). The PCC increased in an age-dependent manner (S4 vs S2). Two months of exercise elevated PCC, in comparison to sedentary animals (E2 vs S2). Conversely, PCC content was significantly reduced in four-month-exercised mice, when compared to age-matched unexercised animals (E4 vs S4). Values were given as means ± std. dev. The level of statistical significance was computed by using two-way ANOVA and post-hoc Newman-Keuls test: *** P<0.001; ** P<0.01. Experiments were performed in triplicate.

The colorimetric assay did not reveal any significant age-dependent change in cortical thiobarbituric acid reactive substances (TBARS) concentrations (Fig. 1a). On the contrary, the content of oxidized proteins increased in an age-dependent way (P<0.001, S4 vs S2) (Fig. 1b). Significant increases in TBARS (Fig. 1a) and protein carbonyl content (PCC) (Fig. 1b) were detected in brain cortices after two months of exercise, when compared to sedentary animals (P<0.001, E2 vs S2). Conversely, four months of exercise did not significantly elevate cortical TBARS levels (Fig. 1a), whereas PCC content was significantly reduced in E4, when compared to S4 (Fig. 1b, P<0.01).

**Figure 2:**
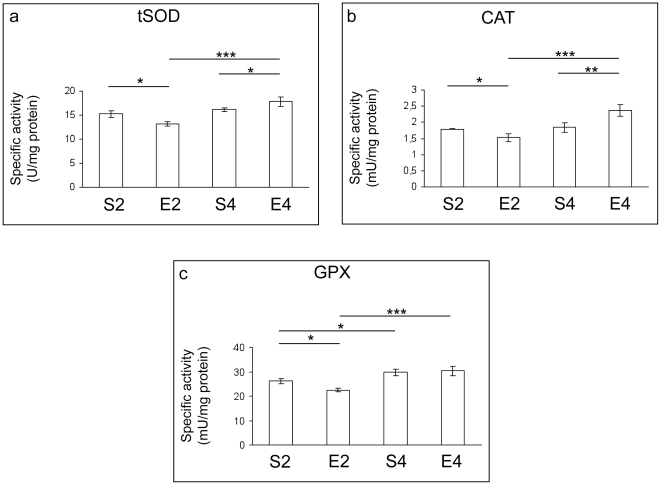
Antioxidant enzymatic defense in brains of mice undergoing a long-term moderate treadmill running. Specific activities of superoxide dismutase (SOD) (panel a), catalase (CAT) (panel b) and glutathione peroxidase (GPX) (panel c) in brain cortices of adult CD1 female mice undergoing a two- or four-month moderate and regular treadmill-based exercise program (E2 or E4, respectively); age-matched sedentary animals (S2, S4) were used as controls (n = 6 per group). No major age-dependent variation of tSOD and CAT specific activities was revealed; however, a significant increase in GPX activity was detected when comparing S4 and S2. Two-month physical activity reduced specific activities of tSOD, CAT and GPX catalytic capacities (E2 vs S2), whereas four-month exercise triggered a significant elevation of tSOD and CAT (E4 vs S4). Values were given as means ± std. dev. The level of statistical significance was computed by using two-way ANOVA and post-hoc Newman-Keuls test: * P<0.05; ** P<0.01; *** P<0.001. Experiments were performed in triplicate.

**Figure 3:**
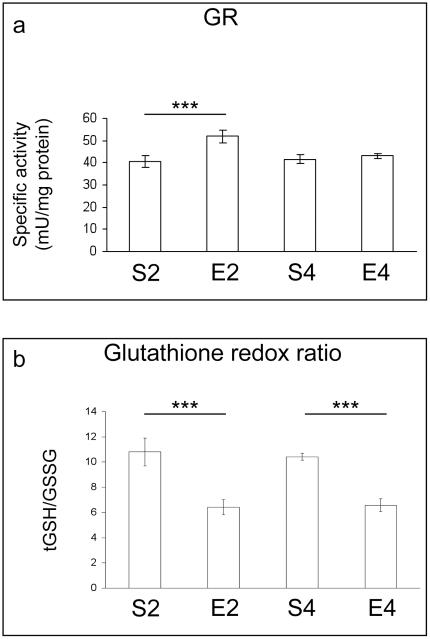
Glutathione-related status in brains of mice undergoing a long-term moderate treadmill running. Specific activity of glutathione reductase (GR) (panel a) and balance of the glutathione redox couple (panel b) in brain cortices of adult CD1 female mice undergoing a two- or four-month moderate and regular treadmill-based exercise program (E2 or E4, respectively); age-matched sedentary animals (S2, S4) were used as controls (n = 6 per group). No significant age-dependent change in GR activity could be observed (S4 vs S2). Two-month physical activity caused a marked increase in GR activity (E2 vs S2), while no statistical differences were found when comparing GR specific activities between E4 and S4 groups. No significant age-dependent change was observed in the total vs oxidized GSH ratio (S4 vs S2). Both two and four months of exercise caused a marked decrease of the total vs disulfide glutathione ratio (E2 vs S2 and E4 vs S4). Values were given as means ± std. dev. The level of statistical significance was computed by using two-way ANOVA and post-hoc Newman-Keuls test: *** P<0.001. Experiments were performed in triplicate.

Spectrophotometric analyses of the catalytic activities of the main antioxidant enzymes revealed no major age-dependent variations of total superoxide dismutase (tSOD) and catalase (CAT) specific activities (Fig. 2a and b), whereas a significant age-dependent increase in GPX activity was observed (P<0.001, S4 vs S2) (Fig. 2c). Significant age-related changes in glutathione reductase (GR) activity could not be detected (Fig. 3a). After two months of regular exercise, marked reductions in brain cortex enzymatic activities of tSOD, CAT and GPX (P<0.05, E2 vs S2) were revealed (Fig. 2a, b and c), together with a marked increase in GR activity (P<0.001, E2 vs S2) (Fig. 3a). On the contrary, four-month exercise caused a significant enhancement of specific activities of tSOD (P<0.05) and CAT (P<0.01), in comparison to the age-matched sedentary group (Fig. 2a and b). No statistical differences were found when comparing GPX and GR specific activities between E4 and S4 (Fig. 2c and 3a).

The colorimetric assessment of the status of the glutathione redox couple revealed no significant age-dependent changes in the cortical total vs oxidized glutathione ratios (Fig. 3b). After both two and four months of exercise, our analysis revealed a remarkable decrease of total vs disulfide glutathione ratios (P<0.001, E2 vs S2 and E4 vs S4) (Fig. 3b).

### Effects of age and exercise on MG-related damage and detoxification system

**Figure 4:**
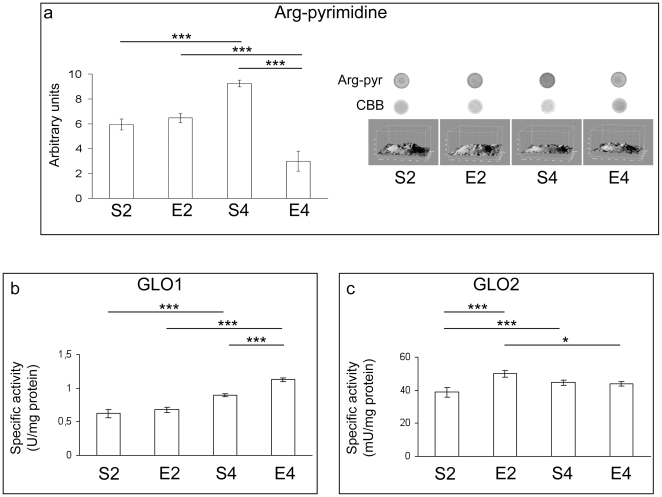
Methylglyoxal-related enzymatic removal and molecular damage in brains of mice undergoing a long-term moderate treadmill running. Immunoreactivity levels against arg-pyrimidine (arg-pyr, panel a) and specific activities of glyoxalase 1 (GLO1, panel b) and glyoxalase 2 (GLO2, panel c) in brain cortices of adult CD1 female mice undergoing a two- or four-month moderate and regular treadmill-based exercise program (E2 or E4, respectively); age-matched sedentary animals (S2, S4) were used as controls (n = 6 per group). Dot blot assays revelead an age-dependent increase in the levels of MG-damaged proteins (S4 vs S2). No significant variation of MG-damaged polypeptides was detected after two months of exercise (E2 vs S2), while immunoreactivity against MG-modified arginine residues decreased markedly after four-month physical activity, with respect to age-matched unexercised mice (E4 vs S4). Immunosignals were normalized against the Coomassie Brilliant Blue (CBB)-based total protein staining. In the panel a, right section, representative PVDF-dot immunoblots of three independent experiments are reported, together with analyses for total protein loading performed by the ImageJ software. Both GLO1 and GLO2 specific activities increased in an age-dependent manner (S4 vs S2). The exercise program elevated GLO2 activity exclusively after two months of regular exercise (E2 vs S2), whereas GLO1 specific activity was increased only after four months of physical activity (E4 vs S4). Values were given as means ± std. dev. The level of statistical significance was computed by using two-way ANOVA and post-hoc Newman-Keuls test: * P<0.05; *** P<0.001. Experiments were performed in triplicate.

The arg-pyrimidine-directed immunoblot analysis showed a very strong age-dependent elevation of the levels of MG-damaged proteins within mice brain cortices (P<0.001, S4 vs S2) (Fig. 4a). Marked age-dependent increases in glyoxalase 1 (GLO1) and glyoxalase 2 (GLO2) specific activities were also observed when comparing S4 to S2 (P<0.001) (Fig. 4b and c). Our analyses showed a slight, but not significant, increase in the levels of MG-damaged proteins within cortices of two-month-exercised mice, together with a striking decrease (P<0.001) of MG-dependent protein damage in brain cortices of four-month-trained mice (Fig. 4a). GLO1 specific activity resulted elevated after four months of running protocol (P<0.001, E4 vs S4), whereas GLO2 catalytic activity was higher as a result of two months of exercise (P<0.001, E2 vs S2) (Fig. 4b and c).

### Effects of age and exercise on BDNF-dependent neurotrophic support

**Figure 5:**
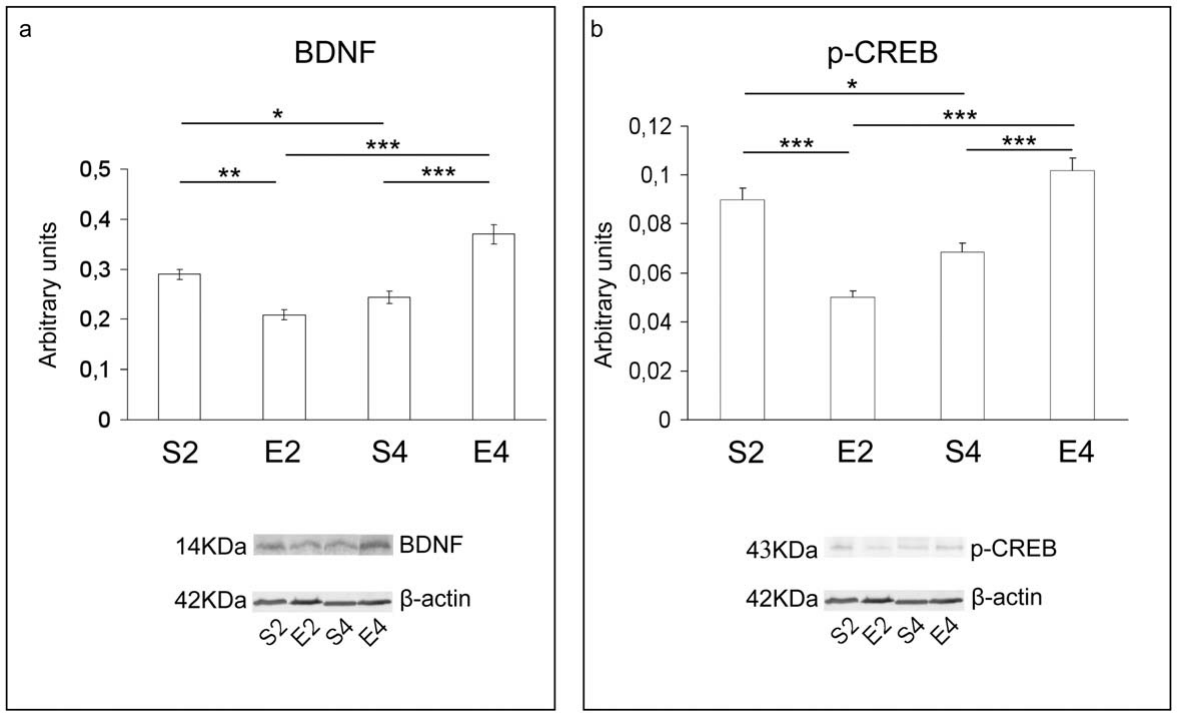
Neurotrophic support in brains of mice undergoing a long-term moderate treadmill running. Western immunoblot against the brain derived neurotrophic factor (BDNF) (panel a) and the phosphorilated cAMP response element binding (p-CREB) transcriptional activator (panel b) in brain cortices of adult CD1 female mice undergoing a two- or four-month moderate and regular treadmill-based exercise program (E2 or E4, respectively); age-matched sedentary animals (S2, S4) were used as controls (n = 6 per group). A significant age-dependent variation in BDNF protein expression was detected (S4 vs S2). BDNF levels were also significantly reduced in two-month exercised mice (E2 vs S2), yet strongly enhanced in four-month exercised mice (E4 vs S4), with respect to age-matched counterparts. Significant age-dependent reductions in the protein levels of p-CREB (S4 vs S2). Two months of exercise caused a conspicuous decrease in p-CREB protein levels (E2 vs S2), whereas p-CREB protein amounts increased after four months of regular running (E4 vs S4), with respect to age-matched unexercised mice. Immunosignals were normalized against the housekeeping β-actin. Representative PVDF-western immunoblots of three independent experiments are reported. Values were given as means ± std. dev. The level of statistical significance was computed by using two-way ANOVA and post-hoc Newman-Keuls test: * P<0.05; ** P<0.01; *** P<0.001. Experiments were performed in triplicate.

**Figure 6:**
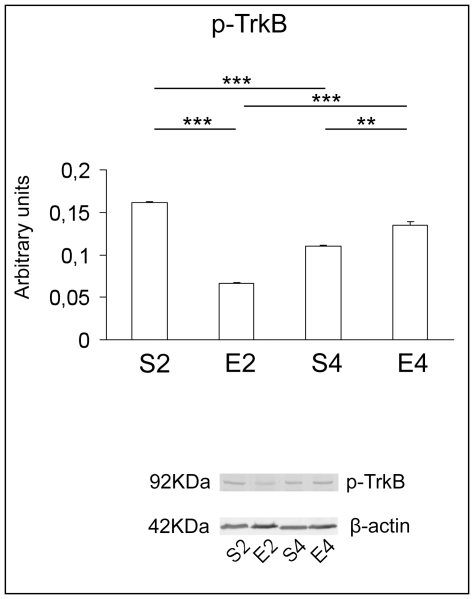
Neurotrophic support in brains of mice undergoing a long-term moderate treadmill running. Western immunoblot against the activated high affinity tyrosinekinase B receptor (p-TrkB) in brain cortices of adult CD1 female mice undergoing a two- or four-month moderate and regular treadmill-based exercise program (E2 or E4, respectively); age-matched sedentary animals (S2, S4) were used as controls (n = 6 per group). Significant age-dependent reductions in the protein amounts of p-TrkB (S4 vs S2) were detected. Two months of exercise caused a conspicuous decrease in the expression levels of p-TrkB, with respect to age-matched unexercised mice (E2 vs S2), whereas protein levels of the activated receptor were significantly elevated after four-month physical activity, when compared to sedentary animals (E4 vs S4). Immunosignals were normalized against the housekeeping β-actin. In the lower sections, representative PVDF-western immunoblots of three independent experiments are reported. Values were given as means ± std. dev. The level of statistical significance was computed by using two-way ANOVA and post-hoc Newman-Keuls test: ** P<0.01; *** P<0.001. Experiments were performed in triplicate.

Significant age-dependent reductions in the protein levels of BDNF, p-CREB (Fig. 5a and b) and p-TrkB (Fig. 6) could be immunodetected in sedentary animals (P<0.05 for BDNF and p-CREB; P<0.001 for p-TrkB). Two months of exercise caused a conspicuous decrease in the protein expression of BDNF (P<0.01), p-CREB (P<0.001) (Fig. 5a and b, respectively) and p-TrkB (P<0.001) (Fig. 6), in comparison with age-matched sedentary mice. In contrast, protein levels of BDNF, p-CREB (Fig. 5a and b) and its activated high affinity receptor (Fig. 6) were significantly elevated after four months of regular running, with respect to age-matched unexercised animals (P<0.001 for both BDNF and p-CREB; P<0.01 for p-TrkB).

## Discussion

Some authors demonstrated that early biomolecular and functional impairments occur in mice brains during the transition from adult to middle age [27], [29]. Coherently, we found important alterations in mouse brain cortices within such time period. In fact, though no major sign of ongoing redox imbalance was detected (Figs. 1a, 2a and b, 3a and b), an age-dependent increase in GPX activity was observed (Fig. 2c). GPX-mediated adaptive responses towards age-related ROS overproduction were previously found by some of us in muscles, livers and lungs of aged rats [32], [33]. Mammalian GPX is known to act as the first line of defence against H_2_O_2_ within cells, having greater affinity for H_2_O_2_ than CAT [34]. Since the ratios of interconvertible forms of redox couples are linked to the cellular redox state, and given the fact that reduced glutathione is an essential co-factor for crucial antioxidant enzymes, the observed lack of age-dependent variations in the total vs oxidized glutathione ratio confirmed that no major redox imbalance occurred within the mouse cortex during the time period investigated. This may imply that antioxidant defense systems and the redox status are adequately efficient and responsive to manage the bland oxidative challenge occurring within brain cortex during the transition from adult to middle age. This could also explain why we did not detect any age-dependent elevation in the levels of lipid peroxidative damage within brain cortex (Fig. 1a), a region particularly prone to free radical attack [5]. Conversely, we found major age-dependent changes in the MG-related protein damage profile, as well as in the specific activities of MG-targeting catabolic enzymes within brain cortices of sedentary animals (Fig. 4a, b and c). MG is one of the most important dicarbonyl precursor of stable advanced glycation end products (AGEs), which are thought to be detrimental factors in the aging process [9], [35]. The formation of AGEs is often associated with the endogenous production of protein carbonyls [36], [37], and this may explain the observed age-dependent elevation of the cortical PCC in sedentary mice (Fig. 1b). Increased protein oxidation is thought to be linked to the age-dependent cerebral functional decline. Indeed, elevated protein carbonyls have been detected in hippocampi of aged rats with memory impairments [38]. Our findings indicated that MG and AGEs may be strictly associated with the transition from adult to middle age within mouse brains, although we revealed an attempt to counteract MG-dependent damages through the activation of the glyoxalase system, which is the main cellular route by which MG is catabolized [9].

In the time period analyzed, significant age-dependent reductions in the protein expression of BDNF, p-CREB (Fig. 5a and b) and activated high affinity TrkB receptor (Fig. 6) were also found in sedentary mice. This enforces the notion that a decline in the activity of the BDNF signaling may be linked to the transition from adult to middle age within brains [39], [40]. Our findings further confirmed the important link between elevated MG-induced molecular damages and the decline in neurotrophic support, as previously suggested by some of us [15].

As a first summary, our data confirmed that the transition from adult to middle age may be a critical biological period in which early hallmarks of tissue senescence can be detected within the mouse brain cortex. The intriguing link between such alterations and the initial age-related decline in cognitive abilities [28] remains to be investigated.

The regular running protocol did not impose excessive stress on the animals, neither did the treatment harm critical health parameters of the animals, as shown by the unchanged ponderal state and appetence behaviour.

Consistently with other reports [41], our findings suggested that one of the hormetic responses triggered in rodent brains by repeated exposures to moderate exercise may be the activation of the major ROS scavenging defense systems. In fact, the four-month exercise program initiated in adult age significantly enhanced the enzymatic activities of important ROS-scavenging systems (SOD and CAT) (Fig. 2a and b). A limited number of studies have investigated the effects of exercise on the major antioxidant enzymatic protection in rodent brains [42], [43]; results are often controversial due to the use of different animal species and exercise paradigms, as well as to the investigation of different biological time periods and CNS regions. Our results are in good accordance with findings reported by Devi and Kiran [43], who demonstrated a high responsivity of CAT and SOD in cortices of adult rats towards the oxidative challenge elicited by 12 weeks swimming training. Our results are not in accordance with data reported by Somani and co-workers [42], who showed unchanged SOD and GPX activities within brain cortices of rodents undergoing long-term running; however, the authors used a different biological model (male rat) and the age of the animals was not even mentioned; in addition, Somani and co-workers [42] used a progressively increasing angle of inclination of the treadmill belt, thus altering the actual work load of the training program, which is a very different type of exercise with respect to that used this study.

As extensively reviewed by Ji [44], most genes coding for antioxidant enzymes contain regulatory sequences in their promoter and intron regions that can interact with redox-sensitive transcription factors, such as the nuclear factor-κB (NF-κB) and activator protein 1 (AP-1). In coherence with the observed enhancement of the major ROS-scavenging systems, the long-term lifestyle intervention inhibited the age-dependent elevation of carbonylated proteins (Fig. 1b). Our findings are not in accordance with results provided by Toldy and co-workers [45]. In their paper, the authors reported no significant changes in brain protein oxidation levels and nuclear factor-κB (NF-κB)/activated protein-1 (AP-1) DNA binding activities in rodents undergoing a training program, in disagreement with the idea of an exercise-induced decrease in SOD activity. However, the experimental design and procedures used were rather different than those we followed. First of all, Toldy and co-workers [45] used a different experimental model (rat). Furthermore, the authors used rodents much younger than our mice, thus exploring a very different time period. Moreover, the exercise protocol was based on a 6-week swimming activity; the last difference appears very important when considering what the authors stated in their discussion (“…running has a more powerful effect than swimming…”) [45].

In accordance with our findings, Radak and colleagues [46] observed that long-term swimming lowered the rat brain content of oxidized proteins through the possible upregulation of the proteasome activity; however, the role played by the proteasome in mediating the training-induced central effects is still largely unclear [30]. The four-month exercise regimen did not cause any significant change in brain TBARS levels (Fig. 1a) and this could imply that, as already discussed, the mouse brain cortex is still adequately protected against the ROS-induced lipid peroxidative damage in the age range under investigation. Our study revealed a remarkable decrease in GSH levels within cortices of four-month exercised animals (Fig. 3b), probably as a consequence of the observed enhancement of the glyoxalase system activity (Fig. 4b), since the reduced form of glutathione is an essential cofactor of GLO1. In coherence with the observed improvement of MG-targeting enzymatic catabolism, we found a remarkable reduction in the levels of MG-modified proteins in cortices of four-month exercised mice (Fig. 4a). To the best of our knowledge, this is the first experimental evidence demonstrating the preventing effect elicited by a habitual and moderate exercise on the age-related accumulation of MG-damaged proteins in mammal CNS. The value of our observation may be worthy of note as elevated amounts of AGEs and MG-modified macromolecules seem to be common hallmarks in the progression of aging, as well as of many age-associated diseases [7], [8].

As already discussed, impaired neurotrophin signaling cascades are known to be linked to aging and to several age-related cognitive dysfunctions [47]. Interestingly, the long-term running markedly improved the neurotrophic support profile within brain cortices. Indeed, we found substantially higher protein expression levels of BDNF, p-CREB (Fig. 5a and b), and p-TrkB (Fig. 6) in four-month treadmill-trained animals, with respect to age-matched sedentary rodents. Other authors suggested that the BDNF-activated pathway could be crucially involved in neuroactive effects of exercise [19], [48]–[51]. Our findings seem to indicate that the BDNF-dependent pathway can still be upregulated within brain cortex even when the long-term exercise is initiated lately in the adult age. Since BDNF is an important modulator of the molecular and enzymatic machinery which protects cells from oxidative stress [52], the upregulated BDNF-dependent signaling may be linked to the increased expression of antioxidant enzymes we observed in four-month-exercised mice, with respect to age-matched sedentary animals.

Taken together, these findings suggested that a four-month moderate exercise program, even when it is initiated in adult age, triggered multiple hormetic responses within the mouse brain cortex through, among others, the activation of molecular machinery aimed at repairing/removing MG-dependent biochemical damages.

Another novel finding arising from this research was that the regular and moderate exercise program elicited a biphasic response in mouse brain cortices. In particular, in two-month exercised mice we found increased cortical levels of TBARS and PCC (Fig. 1a and b), as well as declines in the CAT, SOD and GPX specific activities (Fig. 2a, b and c) and in the levels of reduced GSH (Fig. 3b), with respect to age-matched sedentary mice. These results suggested a redox shift towards a more oxidized cellular environment, in which GSH, the major thiol-based intracellular antioxidant, was strongly challenged by ROS overproduction. This may explain the higher levels of oxidatively-damaged lipids and proteins we found in cortices of two-month-exercised mice (Fig. 1a and b). However, an early tissue responsiveness was observed; in particular, higher specific activities of GR (Fig. 3a) and GLO2 (Fig. 4c) were detected within cortices of two-month-exercised mice, when compared to age-matched unexercised animals. This might indicate the activation of the GSH recycling pathway, which could be most likely triggered by the higher utilization rate of the reduced form of glutathione by the glyoxalase system. The early stimulation of MG-removal system suggested that the increased MG formation may be one of the earliest molecular events occurring in the brain cortex as a result of treadmill running. This, in turn, could contribute to the redox-related biochemical changes we observed, as MG is known to deplete intracellular GSH pool and to facilitate ROS-dependent chemical damage [14], [35], [53]. As a consequence of the induced oxidative stress, the first two months of exercise lowered the cortical protein levels of both BDNF (Fig. 5a) and p-TrkB (Fig. 6), with respect to age-matched sedentary controls. Several mechanisms by which oxidative stress may decrease BDNF have been suggested, including decreased CREB, increased NF-κB DNA-binding activity or energy depletion [54], [55]. Coherently, we found decreased cortical levels of p-CREB in 2-mo exercised animals, with respect to age-matched unexercised controls (Fig. 5b).To the best of our knowledge, this study provides the first experimental evidence of a double-step effect on antioxidative, antiglycative and neurotrophic molecular networks in the cortex of mammals undergoing a long-term, forced and moderate running exercise regimen initiated in adult age. It is not uncommon to observe reduced resistance and resilience in organisms which are already challenged by endogenous processes (e.g., aging, infections, diseases etc) [56]. However, we cannot exclude that transient imbalances in biomolecular networks we investigated in our experimental model might act as stressors required by physical exercise to trigger physiological adaptations and hormetic responses within brains of exercised animals.

In conclusion, our results suggested that a long-term, enforced and moderate exercise initiated in adult age positively affects the major ROS- and MG-targeting scavenging systems and the molecular damage profiles in adult mice brain cortices, preserving as well the BDNF-related neurotrophic support during the physiological transition from adult to middle age.

On the other hand, our findings showed that moderate treadmill running required a time lag in order to activate efficient adaptive mechanisms and compensative adjustments aimed at improving biomolecular redox and neurotrophic patterns within the mouse cortex. Given the multiple systems recruited to modulate the action of physical activity, the understanding of molecular targets underlying exercise-induced physiological adaptations could lead to the development of new therapeutic approaches aimed at retarding cellular senescence and at preventing age-related neurodegenerative diseases. Although many biochemical and molecular aspects still remain to be investigated, this study sheds new interesting lights on the physiological responses and molecular targets activated by regular and moderate physical exercise during the adult and middle ages, thus strengthening the importance of conceiving and developing non-pharmacological approaches aimed at modulating and retarding the brain senescence process.

## Materials and Methods

### Chemicals and antibodies

The monoclonal antibody against arg-pyrimidine was purchased from Cosmo Bio Co., LTD (cat. NOF-N213430-EX; Tokyo, Japan). Rabbit anti-BDNF (cat. sc-20981) and anti-p-CREB (sc-101662) antibodies were supplied by Santa Cruz Biotechnology, Inc. (Santa Cruz, CA, USA). Rabbit anti-TrkB phospho Y705 (cat. ab52191) and anti-β-actin (cat. ab8227) antibodies were purchased from Abcam (Cambridge, UK). The anti-rabbit peroxidase-conjugate secondary antibody (cat. PI-1000) was provided by Vector Laboratories, Inc. (Burlingame, CA, USA). Sigma Aldrich (St. Louis, MO, USA) supplied the anti-mouse secondary antibody (cat. A9044) and all the chemicals, substrates and reagents which are not listed elsewhere. Acrylamide/bis acrylamide (cat. 1610125), premixed Laemmli sample buffer (cat. 1610737), Kaleidoscope prestained standards (cat. 1610324) and Immun-Blot PVDF Sandwiches (cat. 1620239) were purchased from Bio-Rad Laboratories (Hercules, CA, USA). Thermo Fisher Scientific (Rockford, IL, USA) provided metal-enhanced 3′,3-diaminobenzidine (DAB) substrate kit (cat. 34065) and bicinchoninic acid (BCA) protein assay kit (cat. 23225). Cayman Chemical (Ann Arbor, MI,USA) supplied the total/oxidized glutathione (cat. 703002) and the protein carbonyl (cat. 10005020) assay kits. Oxis International (Foster City, CA, USA) provided the colorimetric assay for lipid peroxidation (cat. 21012).

### Animals and running protocol

9-month old CD1 female mice (45–50 grams; N = 48; Harlan Laboratories, Inc., Frederick, MD, USA) were acclimatized for 10 days in the laboratories of the Excellence Research Center on Aging of the University Foundation “G. d'Annunzio” of Chieti (Italy), to housing conditions (22±2°C, 12–12 h light-dark cycle with lights on from 8 a.m. to 8 p.m., free access to water and food, 6 mice per cage). Animals were familiarized to the Exer 3/6 motorized low-noise treadmill (Columbus Instruments, Columbus, OH, USA) for 2 weeks (9 m/min for 10 min, 5 days/week). Rodents were then randomly assigned to four groups (12 mice each) and divided as follows: animals committed to be sacrificed 2 or 4 months later, in presence (E2, E4) or absence (S2, S4) of physical activity protocol.

Exercised groups started the training program (warm-up at 5 m/min for 3 min, running at 13 m/min for 20 min, cool-down at 5 m/min for 3 min, zero inclination, 5 days/week). The final running work load was reached by incrementing 1 minute every day, starting from 10 min/day. Mice were motivated to run by gentle hand prodding, without electric shocks in order to minimize stress. Mice were observed while exercising to ensure continuous running and to monitor signs of undue stress. Sedentary groups were exposed to the same environmental conditions (handling, treadmill motor noise, vibration and deprivation of food and water) while exercising mice performed their running sessions. Food consumption and animal weight were daily monitored throughout the experiment. 24 h after the last training session, mice were rapidly sacrificed by decapitation and cortices were surgically removed and stored at −80°C until further analyses. Every possible effort was taken in order to minimize both the number and the suffering of used animals, according to the principles of the Declaration of Helsinki and to the European Community Council (86/609/CEE). Formal approval for experimental procedures was provided by Health Minister (protocol 13/97-A).

### Sample preparation for enzymatic activity

Brain cortices were homogenized and sonicated (cycle 0.5 – amplitude 50%) in 5 vol of 0.1 M phosphate buffer (pH 7) containing 1 mM ethylenediaminetetraacetic acid (EDTA) and 1.5 mM dithiothreitol (DTT), for subsequent enzymatic analyses of GPX, GLO1, GLO2 and GR, or 0.1% Triton X-100, to assess activities of tSOD and CAT. Then, samples were centrifuged at 15000 g for 30 min at 4°C and supernatants were used for assessments of enzymatic activities and of total protein content [57], by using a Lamba25 spectrophotometer (PerkinElmer Inc., Waltham, MA, USA). Every assay was performed in triplicate.

### Superoxide dismutase

The tSOD (EC 1.15.1.1) activity was assayed by measuring its ability to inhibit the autoxidation of epinephrine, which was spectrophotometrically monitored at 480 nm at 30°C, according to Sun and Zigman [58]. One unit of tSOD activity was assumed to provide 50% of inhibition.

### Catalase

The CAT (EC 1.11.1.6) activity was assayed by monitoring the decomposition of 10 mM H_2_O_2_ at 240 nm, as described by Aebi [59]. One unit of enzyme activity was defined as 1 µmol of H_2_O_2_ reduced/min at 25°C.

### Glutathione peroxidase

The selenium-dependent GPX (EC 1.11.1.9) activity was determined by following the oxidation of nicotinamide adenine dinucleotide phosphate (NADPH) at 340 nm in a glutathione reductase-coupled reaction, using H_2_O_2_ as substrate [60]. One unit of enzyme activity was defined as 1 µmol of GSH-conjugated/min at 25°C.

### Glutathione reductase

The GR (EC 1.6.4.2) activity was monitored by following the conversion of NADPH into NADP^+^ at 340 nm, by using GSSG as substrate [61]. One unit of enzyme activity was defined as 1 µmol of NADPH oxidized/min at 25°C.

### Glyoxalase 1

The GLO1 (EC 4.4.1.5) activity was assayed as described by Mannervik and colleagues [62], by following the production of (R)-S-lactoylglutathione at 240 nm. One unit activity was defined as 1 µmol of lactoylglutathione produced/min at 25°C.

### Glyoxalase 2

The GLO2 (EC 3.1.2.6) activity was assayed by recording the decrease of absorbance at 240 nm [63]. One unit of enzyme activity was defined as 1 µmol of (R)-S-lactoylglutathione hydrolyzed/min at 25°C.

### Western immunoblot

Brain cortices were homogenized and sonicated (cycle 0.5-amplitude 50%) in 5 vol of radio immunoprecipitation assay (RIPA) buffer containing 1% protease inhibitor cocktail and 2% phosphatase inhibitor cocktails I and II. Extracts were centrifuged at 15000 g for 30 min at 4°C and total protein content was assessed by using the BCA method, with bovine serum albumin (BSA) as the standard [64]. Twenty-five micrograms of denatured proteins from each sample were run through polyacrylamide denaturing gels (12–15%) and bands were transferred onto methanol-activated polyvinylidene fluoride (PVDF) sheets by wet electrophoretic transfer [65], [66]. Non-specific binding sites were blocked with 5% (w/v) BSA in Tris-buffered saline containing 0.05% (v/v) Tween 20 (TBS–T) for 1 h. Membranes were then incubated overnight at 4°C with TBS-T containing primary antibodies, by using the following dilution conditions: anti-BDNF (1∶100), anti-p-TrkB (1∶400), anti-p-CREB (1∶75), anti-β-actin (1∶1000). PVDF sheets were washed 3 times with TBS-T (5 min each) and incubated with TBS-T containing the horseradish peroxidase-conjugated secondary antibody (dil. 1∶2000) for 2 h. Membranes were washed 3 times with TBS (5 min each) and the specific immune complexes were detected by using metal-enhanced DAB substrate kit, as suggested by the supplier. Bands were digitally acquired and processed by using the Nonlinear Dynamics TotalLab software. Results were normalized to the signals corresponding to the β-actin housekeeping protein, and given as relative units (RU). The experiments were performed in triplicate.

### Arg-pyrimidine-directed dot immunoblot

Twenty-five micrograms of RIPA-extracted native proteins (see Western immunoblot, sub-section 2.10) were spotted onto methanol-activated PVDF sheets and air dried for 10 min. Membranes were blocked with non-fat dry milk for 1 h and incubated overnight at 4°C with TBS-T containing the monoclonal antibody anti-arg-pyrimidine (dil. 1∶100). Sheets were washed 3 times with TBS-T (5 min each) and incubated with TBS-T containing the horseradish peroxidase-conjugated secondary antibody (dil. 1∶8000) for 2 h. Membranes were washed 3 times with TBS (5 min each) and immune complexes were detected by using the metal-enhanced DAB substrate kit, as recommended by the supplier. Membranes were digitally acquired and data analysis was performed after normalization versus Brilliant Blue Coomassie-R-stained total proteins, by using ImageJ and Nonlinear Dynamics TotalLab softwares [67]. Results were given as relative units (RU). The experiments were performed in triplicate.

### Thiobarbituric acid-reactive substances (TBARS)

The measurement of TBARS is a well-established method used to detect lipid peroxidative damage through the photometric detection of the thiobarbituric acid-conjugated adduct [68]. The final complex was extracted into n-butanol and the absorbance was read at 532 nm by a PerkinElmer Lamba25 spectrophotometer. A linear calibration curve was prepared from pure malondialdehyde (MDA)-containing reactions.

### Protein oxidation

The 2,4-dinitrophenylhydrazine (DNPH)-based assay was used in order to measure the amounts of colorimetrically oxidatively-modified proteins. Extracts obtained as described in sub-section 2.3 were treated to remove nucleic acids by adding 0.1 vol of streptomycin sulphate 10% (w/v), and centrifuged at 6000 g for 10 min at 4°C. 100 µl of supernatants were processed as described by Levine and co-workers [69]. Photometric data at 370 nm were obtained by using a Victor3 microplate reader (PerkinElmer), and normalized to the total protein concentration in the final pellets (absorbance reading at 280 nm; BSA-based linear calibration in the range 0–2.8 mg/ml) in order to consider protein loss during the washing steps, as suggested in the kit's user manual.

### Glutathione redox couple ratio

Total (tGSH) and oxidized glutathione (GSSG) concentrations were measured through a 5,5′-dithiobis-2-nitrobenzoic acid (DTNB)-based enzymatic recycling method [70]. The quantification of GSSG was accomplished by first derivatizing the reduced form of glutathione with 2-vinylpyridine, as recommended by the kit's manufacturer. The absorbance at 405 nm was followed for 30 min by using a Victor3 microplate reader (PerkinElmer). A linear calibration curve was computed from pure GSSG-containing reactions (ranges: 0–8 µM GSSG, 0–16 µM tGSH equivalents).

### Statistical analysis

Statistical analyses were performed by using Statsoft Statistica 7 and SyStat SigmaStat v3.5. Results were given as means ± standard deviations. Ponderal and food uptake profiles were analyzed by 2-way ANOVA for repeated measures. All other dependent variables were processed by 2-way ANOVA analysis, in order to detect main effects due to time, life style and interactions. Post-hoc Newman-Keuls tests were used when appropriate. The null hypothesis was rejected with P<0.05.
